# Utility of High‐Resolution Esophageal Manometry in the Evaluation of Presumed Oropharyngeal Dysphagia

**DOI:** 10.1002/oto2.70168

**Published:** 2025-09-18

**Authors:** David Ahmadian, Phil Tseng, Avin Aggarwal, Austin Lever, Kathleen Cazzato, Helena Yip

**Affiliations:** ^1^ University of Arizona, College of Medicine–Tucson Tucson Arizona USA; ^2^ Department of Internal Medicine University of Arizona, College of Medicine–Tucson, Division of Gastroenterology Tucson Arizona USA; ^3^ Department of Otolaryngology University of Arizona, College of Medicine–Tucson Tucson Arizona USA; ^4^ Department of Speech, Language, and Hearing Sciences University of Arizona Tucson Arizona USA

**Keywords:** clinical statistics, dysphagia/swallowing, upper esophageal sphincter

## Abstract

**Objective:**

Evaluate the utility of high‐resolution esophageal manometry (HREM) in patients with symptoms of oropharyngeal dysphagia (OD) but unremarkable Modified Barium Swallow Studies (MBSS).

**Study Design:**

Retrospective cohort study.

**Setting:**

Tertiary academic center.

**Methods:**

A retrospective review was conducted on patients with symptoms of oropharyngeal dysphagia from January 2021 to December 2024. Demographics, symptoms, and Charlson Comorbidity Index scores were recorded. Patients with unremarkable MBSS then underwent HREM. HREM data based on the Chicago Classification V 3.0 were analyzed, including mean residual upper esophageal sphincter (UES) pressure, median lower esophageal sphincter (LES) pressure, and ineffective swallow rates, using *T*‐tests, and Chi‐Squared tests.

**Results:**

Among 29 patients (mean age 53.2 years, 48.7% female), HREM findings showed mean residual UES pressure of −0.5 mmHg, median residual LES pressure of 13.9 mmHg, and 22.9% ineffective swallows. Esophageal pathologies (ineffective esophageal motility or esophagogastric junction outflow obstruction) were identified in 58.6% of cases. Symptom‐specific differences in esophageal motility disorders were not statistically significant.

**Conclusion:**

HREM identified esophageal pathologies in 58.6% of patients with OD symptoms and normal MBSS, highlighting its value in the diagnostic workup of oropharyngeal dysphagia. The overlap of symptoms between oropharyngeal and esophageal motility disorders supports integrating HREM for comprehensive evaluation.

**Level of Evidence:**

4

Oropharyngeal dysphagia (OD) is a common complaint among patients presenting to the otolaryngology clinic. Its prevalence is estimated to be anywhere between 10% and 22% in US adults ages 50 or older, and between 14.9% and 50.2% in older adults in nursing homes.^1^, ^2^, ^3^ As opposed to esophageal dysphagia, OD involves difficulties in safely and efficiently transferring solids and liquids from the mouth, through the pharynx and upper esophageal sphincter (UES), and into the esophagus.^1^, ^4^ Patients may describe associated symptoms such as a delay in initiation of swallowing, globus sensation, nasal aspiration during swallowing, hoarseness, and weight loss.^1^, ^4^ The etiology of OD is multifactorial but can generally be divided into structural pathologies in the oropharynx, muscular/neuromuscular pathologies, neurodegenerative diseases, and peripheral/central nervous system diseases.^1^, ^4^, ^5^ Validated questionnaires to assess the severity of OD have been developed to study the detrimental impact of OD on patient's quality‐of‐life.^6^, ^7^

The diagnostic workup of OD involves a variety of modalities, the most common of which is a videofluoroscopy (VFS), also known as a Modified Barium Swallow Study (MBSS). VFS/MBSS, is commonly considered to be the gold standard for diagnosis of OD due its real‐time visualization of the transit of a food bolus during swallowing and has been shown to have a sensitivity of 77%, 83%, and 80% for aspiration, penetration, and laryngopharyngeal residues, respectively.^8^ The benefit of MBSS is the ability to assess for all stages of oropharyngeal swallow. There is a brief esophageal screen during an MBSS to help identify potential esophageal abnormalities, but it is not the main focus of the exam. Fiberoptic Endoscopic Evaluation of Swallowing (FEES) complements the use of MBSS in the evaluation of OD. It allows for direct visualization of the oropharynx and may reveal anatomical abnormalities such as obstruction or mass lesions contributing to dysphagia. In addition, FEES is more sensitive for aspiration, penetration, and residue than MBSS.^1^, ^8^, ^9^, ^10^, ^11^, ^12^

OD may occur simultaneously with esophageal dysphagia. In addition, primary esophageal pathology may present with symptoms resembling a pharyngeal dysphagia, making it difficult to establish a clear diagnosis in certain cases.^13^, ^14^, ^15^ Some of the expected causes for dysphagia with OD symptoms and normal MBSS may include muscle tension dysphagia, eosinophilic esophagitis, esophageal stricture, esophageal dysmotility, achalasia of the lower esophageal sphincter (LES), and GERD.^16^ Thus, it is important to localize the site of dysphagia by studies not confined to the pharyngoesophageal junction. Esophageal motility study has evolved since its inception in the 1950s to its modern form of high‐resolution esophageal manometry (HREM) since the early 2000s, to become the gold standard for esophageal motility testing. A catheter with closely spaced pressure sensors is passed into the esophagus and allows for measuring esophageal muscular dynamics, pressure gradients, and motility during swallowing.^17^, ^18^, ^19^ Manometry results are interpreted using the Chicago Classification v3.0 for the past decade, which was developed by an international consensus process to characterize esophageal peristaltic abnormalities. It uses a hierarchical approach, sequentially prioritizing esophageal motility disorders into major and minor categories.^18^, ^19^ Residual LES pressure, which is interchangeable with the term integrated relaxation pressure (IRP), is central in the Chicago Classification v3.0 scheme. Major disorders include achalasia (Type I‐III), defined by ≥15 mmHg and 100% failed peristalsis or spasm; esophagogastric outflow obstruction (EGJOO, defined by median IRP ≥ 15 mmHg and normal esophageal motility); and distal esophageal spasm (DES). Minor disorders include ineffective esophageal motility (IEM) of more than 50% and fragmented peristalsis. The IRP is normal in minor disorders.

Currently, there remains a paucity of data regarding the diagnostic yield of manometry in detecting esophageal pathologies in patients presenting with dysphagia, and OD in particular.^20^, ^21^, ^22^, ^23^, ^24^ In this study, we aimed to characterize the incidence of esophageal pathologies detected using HREM in patients with symptoms suspicious for/suggestive of OD with unremarkable MBSS. Symptoms of OD include direct and indirect symptoms. Direct refers to symptoms such as food sticking in the throat, coughing, and choking. Indirect refers to weight loss, and pneumonia.^25^ The etiologies are neurological, structural, and autoimmune. For our cohort, the symptoms studied were based partially on queries in the two most used clinical swallow assessments, including EAT 10 and Sydney Swallow Questionnaire. Coughing while eating and food lodging are two symptoms tracked on EAT 10 and Sydney Swallow Questionnaire.^26^, ^27^ In addition, choking is a symptom tracked on the Sydney Swallow questionnaire. Patients with GERD can experience dysphagia in the absence of mucosal damage. The American College of Gastroenterology guidelines for patients younger than 50 years of age with dysphagia and no worrisome symptoms call for a 4‐week empiric trial of acid suppression therapy before endoscopy is performed.^28^ Globus has a high association with UES hypertonicity and esophageal motility disorders.^29^ The GERD and globus symptoms in our cohort were related to eating/drinking.

With improved access to HREM studies over the past 2 years at our institution, this advanced diagnostic tool has been integrated into our workup of OD, allowing for a more comprehensive evaluation of patients. The goal of this study was to determine the diagnostic yield of HREM in the workup of patients with clear symptoms of OD but without pathology detected on MBSS.

## Methods

A retrospective chart review investigated patients presenting with OD at a tertiary academic center from January 2021 to December 2024, with approval from the University of Arizona Institutional Review Board. Patients had a diagnosis of dysphagia by ICD‐10 coding at their visit. The cohort of patients referred to the senior author, a laryngologist, all had dysphagia localized to the pharynx. Inclusion criteria required patients to complete both a MBSS and esophageal manometry. These patients were examined via a flexible laryngoscopy by the senior author at their initial visit. All patients with OD underwent an MBSS as part of the workup. MBSS performed at our institution and community hospitals from referring physicians do not share one standard scoring system with clear criteria to denote a normal test. The MBSimp tool is not uniformly employed by speech and language pathologists either. We used the penetration‐aspiration scale score of 1 or a DIGEST score of 0‐1 to define a normal MBSS, as well as not having any physiologic swallowing components identified as abnormal. Similarly, there is no standard protocol at our institution for when FEES is performed. It is based on physician or speech pathologist preference. These patients sometimes have seen the speech pathologist at the outpatient setting before seeing the senior author and therefore a FEES performed at that time. FEES is superior to a flexible laryngoscopy alone in that it not only detects anatomic abnormalities, but also evaluates the function of the oropharynx and hypopharynx during deglutition. There is also the added therapeutic benefit by demonstrating the maneuvers to ensure safe swallows during a FEES.

Demographics (age, gender) and presenting symptoms, including cough while eating, GERD related to eating globus sensation while eating, food lodging, and choking on secretions, were recorded. Symptoms such as dyspnea and dysphonia were excluded due to limited relevance. Categorical variables were summarized with counts and frequencies. Most patients had multiple symptoms, but it was not feasible to divide them into subgroups as these subgroups would overlap and result in many permutations of groups. These subdivisions would weaken the power of analysis. The length of follow‐up was calculated from the initial visit to the last presentation, and comorbidities were scored using the Charlson Comorbidity Index (CCI), which predicts 10‐year survival based on conditions such as heart failure, diabetes, renal disease, or metastatic tumors.^30^

Patients with OD and normal MBSS then underwent HREM. Esophageal manometry reports using Chicago Classification v3.0 contained many data points. We extracted the data regarding mean residual UES pressure, median residual LES pressure, and the frequency of ineffective swallows. In esophageal manometry, residual pressure refers to pressure reading after the esophageal sphincter relaxes, typically during a swallow. This metric is key to determining adequacy of relaxation, identifying outflow obstructions, and diagnosing motility disorders such as achalasia. Median residual LES pressure is synonymous with IRP, which is a measurement of the LES relaxation during swallowing. Specifically, it measures the average lowest pressure within the esophagogastric junction (EGJ) for a 4 seconds period during a 10 seconds window after the UES relaxes when swallowing. Inadequate relaxation denotes achalasia and EGJOO, with an abnormal IRP being >15 mmHg. IRP is central in the hierarchical analysis of the Chicago classification scheme. Major disorders include achalasia which is defined by IRP being abnormal and having 100% failed peristalsis, and EGJ outflow obstruction defined by IRP being abnormal without achalasia. Minor disorders include patients with a normal IRP and >50% ineffective swallows.^31^ The mean UES pressure was recorded but did not figure into the Chicago Classification system. We used the words “mean” for UES pressures and “median” for LES pressures because those were the terms used on manometry reports under the heading of Residual Pressures. The statistical analysis was done with the mean of these values.

Elevated UES and LES pressures were analyzed due to their treatability with botulinum toxin or myotomy, while ineffective swallow frequency was included as a measure of dysmotility with limited treatment options. Continuous variables were summarized as means and standard deviations, and independent *T*‐tests compared patients with and without specific symptoms.

Chicago Classification Version 3 findings from manometry reports were documented to assess rates of ineffective esophageal motility (IEM) and esophagogastric junction outflow obstruction (EGJOO). Chi‐Squared Tests analyzed symptom‐specific differences in classification findings, and p‐values were calculated for comparisons between symptom‐presenting and symptom‐absent patients using *T*‐tests and Chi‐Squared Tests.

## Results

### Patient Characteristics

The selection method yielded a final cohort of 29 patients for data analysis from an initial sample of 203 patients that presented with OD to the senior author's outpatient Laryngology practice. These 29 patients had an unremarkable MBSS initially in the workup for OD, followed by a HREM. The other 174 patients had abnormal MBSS results and were used for comparison based on symptoms. Patients ages ranged from 22 to 82 years old. The average age of patients presenting with dysphagia was 53.2 ± 14.15 years. 48.7% of the cohort was female. 17 patients (58.6%) had an abnormal finding on manometry study as defined by the Chicago Classification V 3.0. The average CCI score for the cohort was 1.72 ± 1.93. Presenting symptoms of the unremarkable MBSS cohort are summarized in Table 1. Among the cohort, 4 patients were referred to Gastroenterology in the author's own institution for Botox injection to the LES, although data on long‐term follow‐up for these patients was unavailable.

**Table 1:** Cohort Clinical Characteristics

	N = 29
Mean age (years)	53.2 (SD = 14.15)
Age range (years)	22‐82
Gender	
Male	10 (34.5%)
Female	19 (48.7%)
Mean CCI	
Presenting symptoms	
Cough	5 (17.2%)
GERD	5 (17.2%)
Globus	8 (27.6%)
Lodged food	22 (75.9%)
Choking	10 (34.5%)
Comorbidities	
Asthma	7 (24.1%)
GERD	18 (62.1%)
Previous PPI use	17 (58.6%)
Smoking history	14 (48.3%)

### Cohort Manometry Findings

Within the study cohort, the average mean residual UES pressure was −0.5 mmHg ± 8.6 mmHg, with normal as being less than 12.0 mmHg. The average median residual LES pressure was 13.9 mmHg ± 5.7 mmHg, with normal being less than 15 mmHg. The mean percent of ineffective swallows was 22.9% ± 30.9%, with ineffective swallow frequency greater than 50% defined as IEM by the Chicago Classification V 3.0. 58.6% of the cohort had esophageal pathologies meeting criteria for IEM and EGJOO, with incidence rates of 10.4% and 48.3%, respectively (Table 2). When stratified by HREM findings, patients with esophageal pathologies had a higher median residual LES pressure (16.96 mmHg) compared to those without esophageal pathologies (9.04 mmHg), and this difference was statistically significant (*P* = .0002). There were no significant differences between mean residual UES pressure and mean percentage of ineffective swallows when stratified by HREM findings.

**Table 2:** Manometry Characteristics in Patients With Dysphagia With Normal MBSS

	N = 29
Mean residual UES (mmHg)	−0.5 (SD = 8.6)
Mean of median residual LES IRP (mmHg)	13.9 (SD = 5.7)
Mean ineffective swallows (%)	22.9% (SD = 30.9)
Chicago classification abnormalities	22 (59.41%)
EGJOO	15 (40.5%)
IEM	5 (13.5%)
Type II achalasia	2 (5.41%)

### Findings by Symptom

The cohort was stratified by presenting symptoms to assess differences in manometric data among patients with and without esophageal pathologies on HREM, but no significant differences were found in symptom rates between the two groups (Table 3).

**Table 3:** Prevalence of Symptomatology in Patients With Abnormal and Normal MBSS

	Abnormal MBSS (N = 174)	Normal MBSS (N = 29)
Cough	23.5%	9.1%
*P*	.33	
GERD	11.8%	18.2%
*P*	.77	
Globus	29.4%	18.2%
*P*	.6	
Lodged food	76.5%	72.7%
*P*	.82	
Choking	41.2%	27.3%
*P*	.45	

Cough while eating was reported by 17.2% of the cohort, with an average mean residual UES pressure of 8.6 ± 15.4 mmHg, median residual LES pressure of 11.4 ± 8.4 mmHg, 32.0% ± 35.6% ineffective swallows, and 60% with EGJ outflow obstruction (EGJOO), ineffective esophageal motility (IEM), or achalasia. Similarly, 17.2% presented with GERD symptoms and dysphagia, with a mean residual UES pressure of −2.0 ± 7.7 mmHg, median residual LES pressure of 13.6 ± 7.0 mmHg, 26.0% ± 37.2% ineffective swallows, and 60% having EGJOO, IEM, or achalasia.

Globus sensation was reported by 27.6% of the cohort, with a mean residual UES pressure of −2.1 ± 2.9 mmHg, median residual LES pressure of 16.4 ± 4.9 mmHg, 16.3% ± 27.7% ineffective swallows, and 75% with EGJOO, IEM, or achalasia. Food lodging was the most common symptom, reported by 75.9% of the cohort, with a mean residual UES pressure of −2.6 ± 4.7 mmHg, median residual LES pressure of 14.9 ± 5.1 mmHg, 22.7% ± 31.3% ineffective swallows, and 61.9% having EGJOO, IEM, or achalasia.

Finally, choking on secretions was reported by 34.5% of the cohort, with a mean residual UES pressure of −1.2 ± 11.6 mmHg, median residual LES pressure of 16.3 ± 5.4 mmHg, 10.0% ± 24.9% ineffective swallows, and 60% with EGJOO, IEM, or achalasia.

### Positive and Negative Symptom Comparison

**Figure 1:**
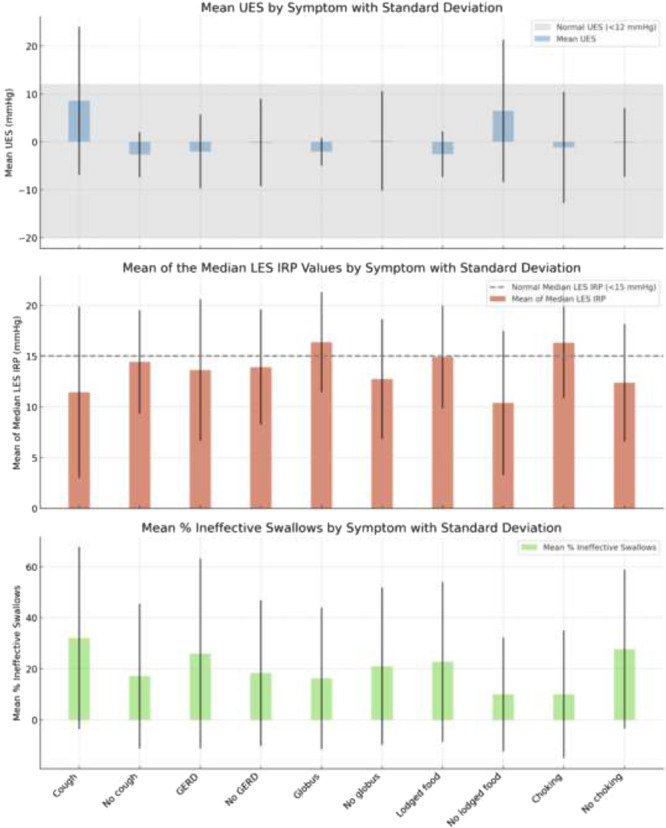
Manometry characteristics stratified by presenting symptoms in patients with dysphagia and normal MBSS in bar graph (illustrating Table 4).

A comparison was made between patients with and without specific symptoms (Table 4 and Figure 1). Figure 1 Manometry characteristics stratified by presenting symptoms in patients with dysphagia and normal MBSS in a bar graph, illustrating Table 4. For patients without cough, the mean residual UES pressure was −2.7 ± 4.7 mmHg, the median residual LES pressure was 14.4 ± 5.1 mmHg, the mean percentage of ineffective swallows was 17.1% ± 28.4%, and 60% of those with cough symptoms had EGJOO, or IEM, or achalasia. A significant difference in mean residual UES pressure was observed between patients with and without cough (*P* = .007).

**Table 4:** Manometry Characteristics Stratified by Presenting Symptoms in Patients With Dysphagia and Normal MBSS

	Mean UES	*P*	Mean of median LES	*P*	Mean % ineffective swallows	*P*	Chicago Classification abnormalities (%)	*P*
Cough	8.56 (SD = 15.43)	.007	11.44 (SD = 8.44)	.31	32 (SD = 35.64)	.31	60	1
No cough	−2.66 (SD = 4.73)		14.43 (SD = 5.10)		17.1 (SD = 28.36)		56.5	
GERD	−2.02 (SD = 7.73)	.67	13.62 (SD = 6.98)	.92	26 (SD = 37.15)	.61	60	1
No GERD	−0.14 (SD = 9.10)		13.91 (SD = 5.66)		18.3 (SD = 28.54)		56.5	
Globus	−2.06 (SD = 2.89)	.55	16.36 (SD = 4.93)	.14	16.3 (SD = 27.74)	.71	75	.41
No globus	0.19 (SD = 10.35)		12.74 (SD = 5.90)		21.0 (SD = 30.81)		52.4	
Lodged food	−2.59 (SD = 4.74)	.023	14.90 (SD = 5.08)	.09	22.7 (SD = 31.35)	.33	61.9	.41
No lodged food	6.45 (SD = 14.89)		10.38 (SD 7.10)		10.0 (SD = 22.36)		43	
Choking	−1.16 (SD = 11.59)	.79	16.31 (SD = 5.44)	.11	10.0 (SD = 24.94)	.21	60	1
No choking	−0.15 (SD = 7.20)		12.37 (SD = 5.81)		27.74 (SD = 31.16)		55.6	

Among patients without GERD symptoms, the mean residual UES pressure was −0.13 ± 9.1 mmHg, the median residual LES pressure was 13.9 ± 5.6 mmHg, the mean percentage of ineffective swallows was 18.3% ± 28.5%, and 60% of those with GERD symptoms had EGJOO, or IEM, or achalasia. Although the mean residual UES and median LES pressures were higher in patients without GERD, the differences were not statistically significant.

For patients without globus sensation, the mean residual UES pressure was 0.19 ± 10.4 mmHg, the median residual LES pressure was 12.7 ± 5.9 mmHg, the mean percentage of ineffective swallows was 21.0% ± 30.8%, and 75% of those with globus symptoms had EGJOO, or IEM, or achalasia. No statistically significant differences in mean residual UES or median LES pressures were observed between groups with and without globus.

Patients without complaints of lodged food had a mean residual UES pressure of 6.5 ± 14.9 mmHg, a median residual LES pressure of 10.4 ± 7.1 mmHg, a mean percentage of ineffective swallows of 10.0% ± 22.4%, and 61.9% of those with lodged food symptoms had EGJOO, or IEM, or achalasia. A significant difference in mean residual UES pressure was noted between the two groups (*P* = .023).

Finally, for patients without choking on secretions, the mean residual UES pressure was −0.2 ± 7.2 mmHg, the median residual LES pressure was 12.4 ± 5.8 mmHg, the mean percentage of ineffective swallows was 26.15% ± 31.2%, and 60% of those with choking symptoms had other abnormalities (Figure 1).

## Discussion

There are many parallels to be drawn between the workup of esophageal and OD. The workup of esophageal dysphagia begins with an upper endoscopy. If that is normal, then the patient is referred for a barium swallow study and HREM. If there are no abnormalities, then the patient is given the diagnosis of functional dysphagia and treated with neuromodulators and behavioral interventions.^32^ Patients with OD also follow a similar diagnostic pathway, but without the HREM. They are usually worked up with a flexible laryngoscopy, and an MBSS, but not necessarily in any order. They may also be referred for a FEES which has the added benefit of evaluating the effectiveness of therapeutic maneuvers and texture modifications.^33^ HREM is not routinely incorporated as part of a workup for OD. When the flexible laryngoscopy, MBSS, and FEES are normal, the diagnosis of muscle tension dysphagia is usually made, where the treatment centers around cognitive behavioral therapy.^34^ This study suggests, however, that this group of patients should be offered additional testing such as HREM to uncover esophageal pathologies. There is a discordance between dysphagia symptoms and localization of the site of swallow dysfunction. Several authors have also found a poor discordance between OD and the actual site of swallow dysfunction including dysmotility and obstruction. Ashraf et al performed a retrospective analysis of 3668 consecutive patients presenting with dysphagia, making it the largest series known to date. 803 patients had an obstructing lesion was seen on upper GI endoscopy or barium swallow study. Of these patients, 48% were accurately able to localize the region of the esophagus contributing to their symptoms. Patients with pharyngeal level lesions were the most accurate in their localization. Conversely, mid and distal esophageal pathology was incorrectly perceived as arising from the throat in 15% to 30% of cases.^15^ Similarly, Madhavan et al performed an analysis of 315 patients with symptoms of “food sticking in the throat” during swallow. They found that 76% of cases had identifiable causes on VFS, with most (71%) being esophageal in origin. Localization accuracy was generally poor, with only 20% of patients who complained of “food sticking in their throat” having explanatory causes identified in the throat (ie, the pharynx or UES), for example.^35^ Roeder et al's analysis also demonstrated a high rate of discordance between patient's perceived localization of dysphagia and the true manometric findings.^36^

Esophageal manometry has been developed to study esophageal peristaltic disorders. Over the last two decades, high‐resolution manometry has replaced conventional manometry because it provides a panoramic and intuitive view of the digestive physiology from the pharynx to the stomach. New investigative parameters were created and esophageal motility disorders reclassified, under the name of Chicago Classification, which has gone through multiple subsequent iterations. Version 3 was a released in 2015 and most recently Version 4 in 2021. Version 4 has more stringent criteria for ineffective motility. In a major departure from CC v3.0, disorders of EGJ outflow and disorders of peristalsis are segregated in CC v4.0, rather than viewed in a hierarchical scheme. The HREM protocol has also been standardized and often necessitates the interpretation of provocative tests including upright swallows, multiple rapid swallows (MRC), and a rapid drink challenge (RDC) in addition to 10 supine swallows. The biggest change of all comes in the characterization of EGJ outflow obstruction (EGJOO) which now requires secondary verification of outflow abnormality with either a timed barium esophagram (TBE/BE) or a functional luminal imaging probe (FLIP) study. Ineffective esophageal motility (IEM) has also been redefined as having more than 70% failed contractions and now grouped with disorders of peristalsis rather than being relegated to a “minor” disorder of peristalsis.^37^

Our study also reveals that a majority of these patient with “food sticking in the throat” but with a normal MBSS may harbor esophageal pathology distant from the primary symptomatic site of the oropharynx. In our cohort of 29 adult patients with unremarkable MBSS, we found that 58.6% had underlying esophageal motility disorders as defined by the Chicago Classification v3. Of these, 48.3% had EGJ outflow obstruction and 10.3% had ineffective esophageal motility. When stratified by esophageal pathologies, patients with abnormal HREM findings had a significantly higher mean residual LES pressure. When stratified by the five major presenting symptoms of OD, the only statistical differences found were in patients with cough on eating, who had a higher UES pressure than patients without cough. Also, patients with food lodged in the throat had a lower UES pressure than ones without. Among the other HREM parameters analyzed, there were no significant differences in patients with and without a given presenting symptom. Similar to findings by Ashraf et al, our study shares the observation that symptoms such as cough with eating, choking, globus, are equivocal in determining whether an esophageal pathology may be present. All of this data supports the notion that no diagnostic modality should be “off the table” when evaluating dysphagia due to a given patient symptom.

EGJ outflow obstruction, defined by IRP of LES > 15 mm Hg, accounted for 82.4% of the esophageal pathologies uncovered in this cohort of patients. Interventions for LES achalasia are similar to those for UES achalasia, comprised of either Botox injection or a myotomy. Ineffective esophageal motility, which has very limited treatment options, only accounted for 17.6% of the esophageal pathologies seen on HREM in this cohort of patients. Traditional diagnostic approaches for OD, such as VFS and FEES, remain mostly qualitative and descriptive in nature. They are limited in studying mostly the upper aerodigestive tract and may miss associated lower esophageal motility abnormalities. Recent advancements in HREM allow for quantifying pressure and motility patterns extending from the UES to LES, greatly expanding the scope of diagnosis. Several studies have demonstrated that HREM can identify motility disorders such as ineffective esophageal motility, achalasia, and gastroesophageal reflux disease (GERD), which may coexist with or contribute to OD.^38^, ^39^

This high incidence of esophageal pathology findings was also found by Laing et al for high‐resolution manometry studies.^40^ The authors examined a series of 855 HREMs, and 53% had esophageal findings including major and minor disorders, EGJOO and achalasia.

In that retrospective analysis, only 14.5% of the HREM met criteria for EGJOO. The clinical significance of various metrics is being continuously revisited by Gastroenterology consensus groups. The Chicago Classification has been revised four times to refine the criteria. V3.0 was designed to eliminate irrelevant metrics. V4.0 further made criteria for ineffective motility more stringent. In v4.0, EGJOO requires a second test of verification including barium tablet or endoFLIP.

Our cohort of patients were analyzed using CCv3.0, and not v4.0 because the latter required a provocative maneuvers protocol during the HREM study as well as a secondary verification method such as endoFLIP which had not been available until December 2024 at our institution. The passage of a barium tablet has not been incorporated as part of our protocol here yet at the time of writing. In a study of 130 patients who had undergone HREM for dysphagia, regurgitation, or non‐cardiac chest pain, the HREM was analyzed using CC v3.0 and v4.0. Motility disorders including achalasia Type I through III and EGJOO remained unchanged in 102 patients. On the other hand, with the more stringent criteria, 38% of the 63 patients diagnosed with IEM on v3.0 were considered to have normal motility on v4.0.^41^

VFSS and FEES are helpful in identifying penetration and aspiration. However, the interpretation of often complex and multifactorial causes of OD remained subjective and mixed in inter‐rater and intra‐rater reliability. As a result, there is growing interest in using pharyngeal HRM for OD in guiding treatment in both swallow rehabilitation and surgical treatment of the UES.^42^ Recent work done by Hoffman et al involving artificial neural networks (ANN) aimed to identify the accuracy of pharyngeal HRM alone in identifying abnormalities detected by the Modified Barium Swallow Impairment Profile (MBSImP), a previously validated tool for quantifying swallow impairment, among 30 patients with dysphagia.^43^, ^44^ The authors found that HRM was approximately 91% accurate when identifying normal or disordered MBSImP parameters and concluded that HREM should supplement and in certain cases replace videofluoroscopic studies. Beyond its diagnostic capabilities, HRM has been shown to have potential utility in the treatment of dysphagia via guiding biofeedback therapy modalities.^26^, ^45^ Although limited to a cohort of 10 patients, Belafsky et al were able to demonstrate significant modulations in UES pressures using HRM‐guided biofeedback, which may provide relief to patients experiencing globus pharyngeus, cricopharyngeal spasm, or dysphagia.^26^ The development of pharyngeal HRM is still in its nascent stage. In 2018, an international working group was convened to develop an expert consensus on the methodology, protocol, and outcome metrics for high‐resolution pharyngeal manometry (HRPM).^46^

UES pressure is recorded on manometry reports but are not used in either Chicago classifications for esophageal dysmotility disorders. Nevertheless, UES pressure would be of particular interest to otolaryngologists and speech and language pathologists. Future studies are necessary to correlate between symptoms, MBSS findings and UES pressures, the presence of a cricopharyngeal bar. For guiding surgical treatment, it would be helpful to see if a cricopharyngeal myotomy could relieve both OD as well as the UES hypertension.

There are important limitations of the present study to consider. Primarily, HREM can provide a wealth of data points and certain anatomical locations with elevated pressures can be targeted for treatment. However, the clinical relevance of the elevated pressures is unclear. Further studies are needed to see if targeted interventions to resolve the elevated pressures will lead to improved patient reported outcomes. Further, this pilot group was limited by its relatively small sample size of 29 patients as well as the retrospective nature of our analysis.

## Conclusion

The workup of dysphagia remains challenging given the often overlapping and ambiguous symptomatology between oropharyngeal and esophageal dysphagia. In the present study, we found that HREM uncovered possible underlying esophageal pathologies in 58.6% of patients with presumed OD and normal MBSS studies. 82% of these patients with esophageal pathologies have EGJOO, which is amenable to procedural interventions.

## Author Contributions

**David Ahmadian**, data curation, data analysis, investigation, writing—review and editing; **Phil Tseng**, data collection, data analysis, investigation, writing—original draft; **Avin Aggarwal**, methodology, writing—review and editing; **Austin Lever**, methodology, study design conception; **Kathleen Cazzato**, methodology, writing—review and editing; **Helena Yip**, conceptualization, data curation, methodology, project administration, supervision, validation, writing—review and editing.

## Disclosures

### Competing interests

None.

### Funding source

None.

## Ethics Statement

This study was conducted at the University of Arizona College of Medicine – Tucson and approved by the Institutional Review Board of the University of Arizona (IRB #STUDY00003289).

## Data Availability

The data that support the findings of this study are available on request from the corresponding author. The data are not publicly available due to privacy or ethical restrictions.
